# Diagnostic performance of non-contrast quiescent-interval slice-selective (QISS) magnetic resonance angiography for evaluation of the renal arterial vasculature compared to computed tomography angiography (CTA) as reference standard

**DOI:** 10.1016/j.ejro.2025.100706

**Published:** 2025-11-08

**Authors:** Patrick Ghibes, Florian Hagen, Petros Martirosian, Stephan Ursprung, Konstantin Nikolaou, Daniel Raskin, Abraham Levitin, Levester Kirksey, Sasan Partovi

**Affiliations:** a Department for Diagnostic and Interventional Radiology, University Hospital Tuebingen, Tuebingen, Germany; b Section on Experimental Radiology, Department of Diagnostic and Interventional Radiology, University Hospital Tuebingen, Tuebingen, Germany; c Interventional Radiology, Cleveland Clinic Main Campus, Cleveland, OH, United States; d Vascular Surgery, Cleveland Clinic Main Campus, Cleveland, OH, United States

**Keywords:** QISS MRA, CTA, Renal arterial vasculature, Renal artery branching patterns

## Abstract

**Purpose:**

To evaluate the diagnostic quality and detection of anatomical variants in branching patterns of the renal arteries for non-contrast quiescent-interval slice-selective (QISS) MR Angiography (MRA) compared to CT Angiography (CTA).

**Methods:**

Patients who underwent a QISS MRA of the renal arteries as well as CTA as reference standard were included in this retrospective study. Signal-to-noise ratio (SNR), contrast-to-noise ratio (SNR), and vessel diameter were determined in the left and right renal arterial systems. Image quality and diagnostic confidence were assessed with a standardized five-point Likert scale. Sensitivity, specificity and accuracy for the detection of anatomical variants in branching patterns (accessory renal artery, aberrant renal artery and early branching) of the renal arterial system were determined compared to CTA as reference standard.

**Results:**

30 patients (59 renal arteries) were included in this retrospective study. CTA reached significantly higher median SNR compared to QISS MRA (10.96, inter-quartile range (IQR) 6.70–16.11 vs. 5.65, IQR 4.38–8.76, respectively, p < 0.001). Median CNR was significantly higher in QISS MRA (16.75, IQR 13.09–20.96) compared to CTA (13.22, IQR 7.49–18.57), p = 0.006. Diameters of the renal arteries were similar between QISS MRA and CTA (5.8 mm, IQR 4.90–6.60 versus 5.8 mm, IQR 4.75, 6.70, p = 0.893). Diagnostic confidence was rated excellent for both, though significantly higher for CTA (5, IQR 5–5,) compared to QISS MRA (5, IQR 4–5), p = 0.003). 19 of 20 variants in branching pattern could be detected successfully by QISS.

**Conclusion:**

QISS MRA offers similar diagnostic confidence and image quality to CTA as reference standard. Further, QISS MRA demonstrates excellent diagnostic accuracy in detecting anatomical variants of branching patterns of the renal arterial vasculature.

## 1 Introduction

Imaging of the renal arterial vasculature is of paramount importance in daily clinical practice for diagnosing and managing a variety of renal artery diseases, such as atherosclerosis related stenotic disease or fibromuscular dysplasia. Further, the continuously rising number of living kidney donors requires precise imaging of the renal arterial vasculature in healthy subjects prior to consideration for serving as donors for renal transplantation [1]. Anatomical variations in vascular patterns have important implications for pre-procedural planning and may represent a contraindication for living kidney donors [2]. Detailed assessment of the renal arterial vasculature is also important in patients with therapy refractory hypertension [3].

A variety of studies have investigated the incidence of anatomical variations with regard to renal arterial vasculature branching patterns and significant differences between different study collectives were found. Arterial supply by a singular renal artery can only be expected in 63–95 % of patients, revealing the critical role of correctly assessing the renal arterial vasculature [4].

Due to its wide availability and rapid image acquisition, computed tomography (CT) has become the imaging modality of choice for assessment of the renal arterial vasculature. Current multidetector CT angiography (CTA) enables precise evaluation of abdominal vascular pathologies [5].

Despite the high diagnostic accuracy, the suitability of CTA is limited in certain patient populations. Radiation exposure can be a relative contraindication in young patients or healthy living donors [6]. Impaired renal function may be considered as a contraindication for CTA due to the administration of nephrotoxic iodine-based contrast agents [2].

Contrast-enhanced magnetic resonance angiography (CE-MRA) is an ionizing radiation-free alternative to CTA for accurately assessing the renal arterial vasculature, although allergic reactions to Gadolinium based contrast agents may limit the administration in affected patients [7].

Non-contrast MRI angiography (MRA) techniques are less invasive without the requirement for gadolinium-based contrast administration and provide an equivalent option for vascular imaging of a variety of disease processes [8]. Different non-contrast MRA sequences are currently available including time-of-flight (TOF) angiography, subtractive three-dimensional fast-spin echo or balanced steady-state free precession techniques (bSSFP). However, the use of non-contrast MRA approaches in clinical routine is limited secondary to the lengthy measurement times and significant motions artifacts [9]. A further development in the field of non-constrast MRA is the so-called Quiescent interval single-shot (QISS) sequence. The main focus during the development of this sequence was to have a robust and reliable technique suitable for routine clinical applications. QISS MR angiography (MRA) uses a modified single shot two-dimensional (bSSFP) pulse sequence and allows faster acquisition with decreased motion artifacts and excellent visualization of the arterial vasculature [10]. QISS MRA has been primarily investigated for the diagnosis and management of peripheral artery disease (PAD) [11]. A very limited number of studies have applied QISS MRA for imaging of the abdominal arterial vasculature including the renal arteries Andersson et al. investigated the use of QISS MRA in healthy potential kidney donors for the visualization of the renal vasculature in comparison to contrast-enhanced MRA [12]. Another study by Serhal et al. demonstrated that QISS MRA may serve as a potential unenhanced MR approach for the visualization of vascular anastomoses status post renal transplant [13]. Edelman et al. published a study in 2020 in which an isotropic QISS MRA sequence was used to depict the renal arterial vasculature in seven patients [9].

To the best of our knowledge QISS MRA of the renal arterial vasculature has only be evaluated in small cohorts consisting primarily of healthy volunteers and was not compared thus far to standard of care imaging modalities, such as CTA or digital-subtraction angiography (DSA). The aim of this study is to investigate QISS-MRA as a non-contrast MRA approach for the evaluation of the renal arterial vasculature with regard to diagnostic image quality as well as diagnostic accuracy in detecting anatomical variations in branching patterns of the renal arterial vasculature compared to CTA as reference standard.

## 2 Material and methods

### 2.1 Subjects

Between October and December 2024, consecutive patients who underwent MRI of the abdomen including QISS MRA of the renal arterial vasculature with previously performed CTA were included in this retrospective analysis. This retrospective HIPAA-compliant study was approved by the local institutional review board (approval number 297/2023BO2). The requirement for informed consent was waived.

Inclusion criteria were (a) liver disease, kidney disease or other abdominal pathology with indication for abdominal MRI, (b) CTA of the abdomen including coverage of the renal arterial vasculature and (c) QISS MRA of the abdomen visualizing the renal vasculature.

Exclusion criteria were (a) history of endovascular therapy or surgery affecting the renal arterial vasculature prior to conducting QISS MRA and (b) non-diagnostic image quality of the CTA due to artifacts.

### 2.2 Image acquisition

#### 2.2.1 QISS MRA protocol

QISS MRA was performed on a 1.5 T MRI scanner (Sola, Siemens Healthineers AG, Erlangen, Germany) with subjects positioned supine with feet first using 32-channel spine array and 18-channel body array coils. All patients underwent QISS MRA via acquisition of the bSSFP sequence. Measurements were performed during breath-holding and pulse triggering. Spatial resolution was 0.5 mm× 0.5 mm and the slice thickness was 3 mm. The field-of-view (FOV) was positioned with the cranial boundary approximately 2 cm above the diaphragm and the caudal boundary approximately 2 cm below the kidneys. The number of slices was selected to cover the whole renal arterial vasculature from the origin at the abdominal aorta and the entire kidney, thereby visualizing the branching pattern of the renal arterial vasculature including second and third order branches. Images were acquired separately for the left and right kidney in sagittal orientation with flow adjusted to the renal arterial vasculature while pursuing venous flow saturation of the renal venous system.

#### 2.2.2 CTA protocol

The patients underwent CTA of the abdomen using various CT scanners: first generation spectral photon-counting scanner (NAEOTOM Alpha, Siemens Healthineers, Erlangen, Germany), third generation dual-energy energy-integrating detector (EID) CT scanner (Force, Siemens Healthineers, Erlangen, Germany) or a single source EID CT scanner (Somatom X.cite, Siemens Healthineers, Erlangen, Germany). Single source modus was used for the dual-energy scanner. The imaging protocol was standardized across all scanners (see Table 2). The CTA image acquisition was initiated based on bolus tracking. The field-of-view (FOV) was positioned with the cranial boundary approximately 2 cm above the diaphragm and the caudal boundary approximately at the height of the iliac spine or the femoral head. The iodine-based contrast agent (Ultravist 370, Bayer Schering Pharma, Berlin, Germany) was administered intravenously dosed by the patient’s body weight utilizing an automated power injector (60–100 mL, flow 2.5–3 mL/s), followed by a saline flush (50–80 mL, 2.5–3 mL/s). The region of interest (ROI) for bolus tracking was placed in the abdominal aorta at the level of the celiac trunk. The CT acquisition was started as soon as the CT value in the ROI reached 200 HU. The matrix size was 512 × 512 for all scanners. Images were reconstructed with a slice thickness of 1 mm and 3 mm for axial images as well as with a slice thickness of 3 mm for coronal images.

### 2.3 Image analysis

#### 2.3.1 Objective image analysis

For objective image evaluation the Signal-to-noise ratio (SNR) and the Contrast-to-noise ratio (CNR) for QISS MRA and CTA were determined. To this end, ROIs were positioned in the proximal left and right renal artery. Additional ROIs were placed in the psoas musculature at the level of the renal artery. The SNR and CNR were calculated applying the following equations:

**SNR**_**CTA**_**= (CT-value [HU**_**ROI**_**] / Noise [SD**_**ROI**_**])**

**SNR**_**MRA**_**= (Mean signal**_**ROI**_**/ SD**_**ROI**_**)**

**CNR**_**CTA**_**= (CT-value [HU**_**ROI**_**] – CT-value [HU**_**muscle**_**]) / Noise [SD**_**muscle**_**] CNR**_**MRA**_**= (Mean signal**_**ROI**_**– Mean signal**_**muscle**_**) / SD**_**muscle**_

The presence of anatomical variations of the renal arterial vasculature were assessed for QISS MRA and CTA. Renal artery variations were defined as: normal; accessory renal artery; early branching (1.5 cm from the origin for the left renal artery or in the retrocaval segment for the right renal artery); aberrant renal arteries.

#### 2.3.2 Subjective image analysis

Two radiologists with five and six years of experience in vascular imaging assessed the image quality independently, using blinded random order reading of QISS MRA and CTA datasets side-by-side. Differences in image quality rating between the two readers were resolved by a dedicated consensus reading. Image quality assessment included the subcategories diagnostic confidence, vessel contrast and noise. Each parameter was assessed using a modified Likert scale (1: images non-diagnostic”; 2: ‘poor’ defined as “major artifacts/ presence of poor vessel contrast and, therefore, clinical use is not advised”; 3: ‘average’ defined as “borderline clinical use due to the image quality and, hence, borderline diagnostic”; 4: ‘good’ defined as “containing minor artifacts and good vessel contrast which do not adversely affect the clinical usage and these images are, therefore, diagnostic”; and 5: ‘excellent’ defined as “free of artifacts and fully diagnostic”). The readers also evaluated image noise using the following five-point Likert scale: 1 = severe noise, 2 = moderate noise, 3 = mild noise, 4 = minimal noise, 5 = lack of noise.

### 2.4 Statistical analysis

Statistical analyses were performed using SPSS 28 (IBM Corporation, Armonk, NY).

SNR and CNR of QISS MRA and CTA images were compared for each vessel segment individually using Friedman test and a paired Wilcoxon signed-rank test for post-hoc analysis. P < 0.05 was considered statistically significant.

The reading scores for qualitative image analysis of the QISS MRA and CTA were compared for each vessel segment individually using Friedman test and a paired Wilcoxon signed-rank test for post-hoc analysis. P < 0.05 was considered statistically significant.

To compare the diagnostic performance of QISS MRA to CTA, sensitivity, specificity and accuracy were calculated for the left and right renal artery with regard to the detection of anatomical variations in branching patterns of the renal arterial vasculature.

## 3 Results

Between October and December 2024, 30 patients (mean age 74.68 ± 11.10 years), who had previously undergone CTA of the renal vasculature, underwent QISS MRA of the renal arteries. None of these patients had to be excluded from the study. Patients’ baseline characteristics are summarized in Table 1. The indication for MRI and CT was mostly (n = 22) related to underlaying malignancy or metastatic disease to the liver. Mean QISS MRA measurement time for the right renal artery was 2 min 12 s ± 90 s and for the left renal artery was 3 min 22 s ± 47 s. One patient had previously undergone right-sided nephrectomy, hence a total of 59 renal arteries were identified and analyzed. All included vascular segments were classified as diagnostic, hence no segment had to be excluded from the final analysis.

**Table 1:** Baseline characteristics of the investigated patient population.

Characteristics	All participants (n = 30)
Male	20 (67 %)
Female	10 (33 %)
Mean age [years]*	74.68 ± 11.10
Body mass index [kg/m^2^]*	25.97 ± 4.07
Cardiovascular disease risk factors	
Hypertension	13 (43 %)
Dyslipidemia	7 (23 %)
Diabetes mellitus	5 (17 %)
Coronary heart disease	6 (20 %)
Smoking history	4 (13 %)

Note: Unless otherwise indicated, data area numbers of patients with percentage in parentheses. * Data are means ± SDs.

### 3.1 Quantitative analysis

The evaluation of SNR revealed significantly higher SNR for CTA compared to QISS MRA for the left and the right renal artery (see Table 3). SNR was 10.96 (IQR 6.70–16.11) for CTA and 5.65 (IQR 4.38–8.76) for QISS MRA, (p < 0.001). The evaluation of the CTA images revealed a significantly higher SNR of the right (14.29, IQR 9.38–20.00) compared to the left renal artery (9.12, IQR 5.99–13.39), p = 0.015. No significant differences were found in QISS MRA between the right and the left renal arteries.

**Table 2:** Technical CT exam parameters.

Parameter	Naetom Alpha	Somatom Force	Somatom X.cite
Tube potential (kVp)	140	90	120
Matrix	512 × 512	512 × 512	512 × 512
Slice thickness (mm)	3	3	3
Reconstruction kernel	Br40	Br40	Br40
Iterative reconstruction algorithm	QIR3	ADMIRE3	ADMIRE3

**Table 3:** Objective image parameters including SNR, CNR, noise and diameter of the renal arterial vasculature on QISS MRA compared to CTA as reference standard.

	CTA	QISS MRA	p-value
**SNR**			
Bilateral	10.96 (6.70, 16.11)	5.65 (4.38, 8.76)	< 0.001*
Right	14.29 (9.38,20.00)	5.89 (4.22, 7.64)	< 0.001*
Left	9.12 (5.99, 13.39)	5.63 (5.99, 13.39)	0.004*
**CNR**			
Bilateral	13.22 (7.49, 18.57)	16.75 (13.09, 20.96)	0.006*
Right	12.17 (7.27, 18.37)	18.69 (13.96, 22.40)	0.032*
Left	13.23 (7.39, 18.37)	14.82 (12.49, 18.71)	0.090
**Diameter renal artery**			
Overall	5.80 (4.90, 6.60)	5.80 (4.75, 6.70)	0.893
Right	5.70 (4.90, 6.60)	5.60 (4.70, 6.40)	0.852
Left	5.90 (5.00, 6.57)	6.00 (4.85, 6.75)	0,668

Median, IQR and p-values are listed for CTA and QISS MRA.

CNR was increased for QISS MRA compared to CTA for the right and left renal artery, however this finding was solely significant for the right renal artery (18.69 (IQR 13.96–22.40) for QISS MRA and 12.17 (IQR 7.27–18.37) for CTA, p = 0.032).

No significant differences were found for the diameter of the renal arterial vasculature. The median diameter of the bilateral renal arteries was 5.80 (IQR 4.90–6.60) for CTA and 5.80 (IQR 4.75–6.70) for QISS MRA, p = 0.893.

### 3.2 Qualitative evaluation

Qualitative image analysis (see Table 4) revealed excellent diagnostic confidence for QISS MRA and CTA, however the rating was higher for CTA (5, IQR 5–5) compared to QISS MRA (5, IQR 4–5) (p = 0.003).

**Table 4:** Image quality rating with regard to diagnostic confidence, vessel contrast and noise.

	CTA	QISS MRA	p-value
**Diagnostic Confidence**			
Bilateral	5 (5,5)	5 (4,5)	0.003*
Right	5 (5,5)	5 (4,5)	0.041*
Left	5 (5,5)	5 (4,5)	0.029*
**Vessel Contrast**			
Bilateral	5 (4,5)	4 (4,5)	0.446
Right	5 (4,5)	4 (4,5)	0.986
Left	4.5 (4,5)	4 (4,5)	0.280
**Noise**			
Bilateral	5 (4,5)	4 (3,4)	< 0.001*
Right	5 (4,5)	4 (3,4)	< 0.001*
Left	5 (4,5)	4 (3,4)	< 0.001*

Median, IQR and p-values are listed for CTA and QISS MRA.

No significant differences were found for vessel contrast, e.g. for the right renal artery between CTA (5; IQR 4–5) and QISS MRA (4; IQR 4–5; p = 0.986).

Regarding the noise rating, CTA revealed no noise (5, IQR 4–5), however minimal noise (4, IQR 4–5) was detected in QISS MRA (p = 0.001).

### 3.3 Diagnostic performance of renal vascular branching pattern detection

**Fig. 1:**
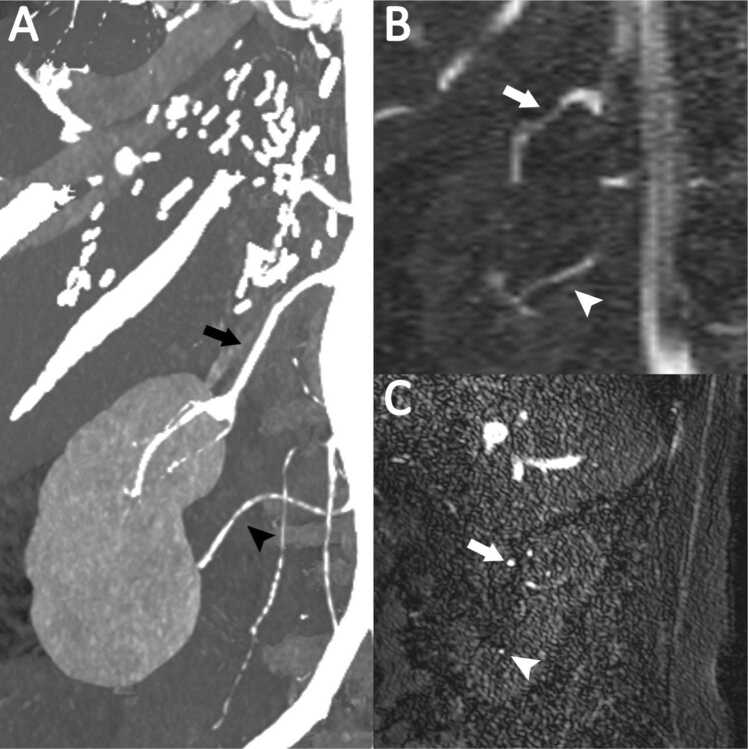
72 year-old female with aberrant right renal artery. (A) MIP Reconstruction of Photon-Counting CTA demonstrating main renal artery supplying the superior pole (arrow) and aberrant renal artery supplying the inferior pole (arrowhead). Main renal artery (arrow) and aberrant renal artery (arrowhead) are also visible in the QISS MRA coronal MIP (B) and the corresponding QISS MRA sagittal slices (C).

**Fig. 2:**
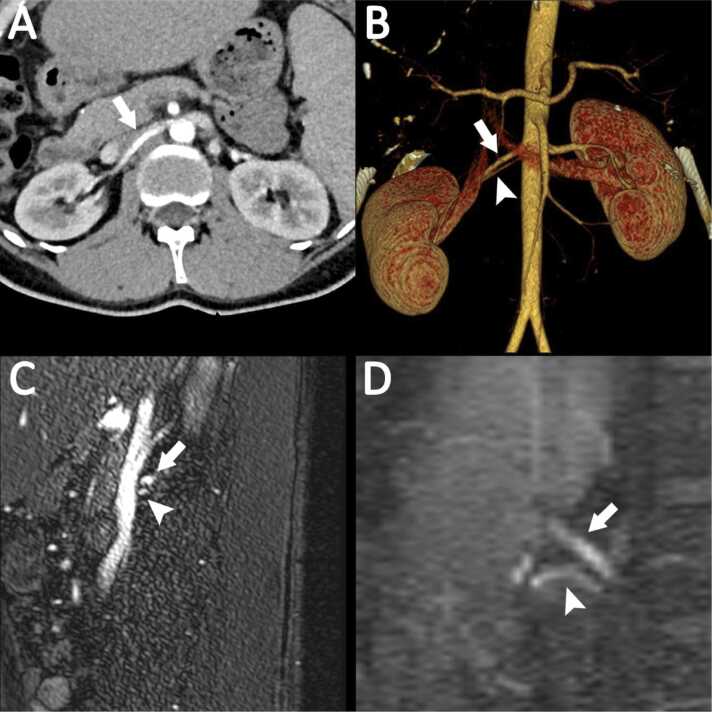
76 year-old male with accessory right renal artery coursing underneath the main renal artery (arrow), which is not clearly detectable on axial slices of the dual-energy CTA (A), however visible (arrowhead) on the coronal MIP CTA reconstruction (B). Right renal artery (arrow) and accessory renal artery (arrowhead) are detected reliably on sagittal slices of QISS MRA (C) as well as on the QISS MRA coronal acquisitions (D).

In 30 patients a total of 20 anatomical variations of branching pattern were detected by CTA. Fig. 1, Fig. 2 demonstrate representative examples of anatomical variations in branching pattern. QISS MRA was able to detect 19 of the 20 variations (95 %) in the renal arterial vasculature branching patterns. One early branching of the left renal artery could not be detected by QISS MRA due to incorrect estimation of the distance between the origin of the left renal artery and the branching of the renal arterial vasculature on sagittal slices. No additional (false) branching pattern was found by QISS MRA. Sensitivity, specificity and accuracy for detection of an accessory renal artery and an aberrant renal artery was 100 %, 100 % and 100 %, respectively, for QISS MRA. For QISS MRA sensitivity for detection of early branching was 89 %, specificity was 100 % and accuracy was 93 %. The diagnostic performance data are listed in Table 5.

**Table 5:** Diagnostic performance of QISS MRA versus reference standard CTA. Sensitivity, specificity and accuracy are listed for the investigated anatomical variations of the renal arterial vasculature.

Vascular anomaly	Cases (n)	Sensitivity [%]	Specificity [%]	Accuracy [%]
**Accessory renal artery**				
Bilateral	7	100 (59, 100)	100 (93, 100)	100(94, 100)
Right	3	100 (29, 100)	100 (87, 100)	100 (88, 100)
Left	4	100 (29, 100)	100 (87, 100)	100 (88, 100)
**Abberant renal artery**				
Bilateral	4	100 (40, 100)	100 (94, 100)	100 (94, 100)
Right	2	100 (16, 100)	100 (87, 100)	100 (88, 100)
Left	2	100 (16, 100)	100 (88, 100)	100 (88, 100)
**Early branching**				
Bilateral	9	89 (52, 100)	100 (93,100)	98 (91, 100)
Right	4	100 (40, 100)	100 (86, 100)	100 (88, 100)
Left	5	80 (28, 99)	100 (86, 100)	97 (83, 100)

Data in parentheses are 95 % confidence intervals.

## 4 Discussion

QISS non-enhanced MRA is an evolving MR imaging technology in clinical routine, which offers the possibility of vascular imaging without need for gadolinium-based contrast agent administration and without ionizing radiation exposure. This study demonstrated that QISS non-enhanced MRA enables reliable visualization of anatomical variations in branching patterns of the renal arterial vasculature with a high image quality and diagnostic confidence. Despite its lower SNR, QISS MRA achieved an increased CNR and highly accurate visualization of vascular diameters.

Quantitative analysis was performed via SNR and CNR. QISS MRA revealed lower SNR compared to CTA. One reason for this may relate to the fact, that bSSFP pulse sequences are sensitive for magnetic susceptibility-related artifacts, which are common in abdominal imaging and may cause noise intravascularly and in the surrounding tissue [11]. Alternatively, fast low-angle shot (FLASH) QISS sequences may be applied, which are known to generate lower intravascular signal and require lengthier acquisition times [14], [15]. For elderly patients and MR sequences requiring breath holding, measurement time can become the limiting factor of compliance and successful acquisition of MR images. Therefore, in this study we opted to apply bSSFP sequences [16]. Another possible reason for lower SNR of QISS MRA compared to CTA is the usage of pulse gating, which can cause a loss of intravascular signal [10]. Pulse gating was used to minimize the preparation time of patients. Furthermore, based on the institutional experience pulse triggering is more reliable compared to ECG gating approaches.

CNR of QISS MRA was superior compared to CTA. This underlines the advantage of the QISS sequences since the signal of surrounding tissue is suppressed while high intraarterial signal is generated. Subjective image parameters were rated good to excellent, albeit slightly lower, in QISS MRA compared to CTA, rendering QISS MRA suitable for vascular diagnostics in daily clinical practice. Importantly, the visual impression of the intravascular signal of QISS MRA is not necessarily comparable to CTA, hence readers need to have experience with MRA to achieve accurate interpretation of QISS MRA for renal arterial vasculature assessment [17]. While CTA requires administration of contrast medium, one of the major advantages of QISS MRA is the unenhanced approach, leading to superior intravascular signal compared to early-phase contrast-enhanced MR and CT images. Early enhancement of the renal tissue as well as surrounding arteries and veins can make it challenging to visualize and delineate small peripheral renal arteries or accessory renal arterial vessels [18], [19]. QISS MRA generate purely intravascular signal, independent from the surrounding tissues and, therefore, hilar as well as intraparenchymal arteries have high SNR and CNR.

Variations in branching patterns were found in this study in a total of 15 patients, demonstrating the importance of adequate imaging of the renal vasculature. The prevalence of 50 % for presence of branching patterns is similar to previously published studies, for example Munnusamy et al. revealed an incidence of 51 % in their study [20]. Accessory renal arteries were found in 20 % of our patients and this prevalence is in the same range of studies with larger patient collectives [21], [22]. QISS MRA was able to reliably detect additional renal arteries as well as aberrant and early branching patterns. The sagittal acquired images in QUISS MRA were found to be particularly helpful for assessment of the renal arterial vasculature. Renal arteries originate from the aorta and run usually orthogonally to the renal hilus in sagittal slices. The high spatial resolution as well as the possibility of suppression of venous vessels enables precise visualization of even the small renal arterial vessels. In the clinical setting, this information can be essential for preoperative imaging in potential living kidney donors, in whom even the smallest aberrant or accessory renal vessel must be correctly identified presurgically [23]. Of note, for renal artery stenosis evaluation sagittal slices are considered suboptimal. Despite respiratory triggering, motion artifacts can mimic or mask renal artery stenosis on sagittal images. Fischer et al. utilized coronal or oblique coronal slices in their study, hence renal arteries and associated stenotic disease can be visualized in one image slice without motion artifacts [12]. QISS MRA allows suppressing venous vascular signal thanks to the opposed flow direction compared to arterial vessels. This technical feature contributes to high sensitivity and specificity in detecting anatomical variations in the renal arterial vasculature branching patterns, as shown in this and a recently published study [12]. Lal et al. describe the difficulty to detect small arterial vessels using bSSFP sequences due to background noise [24]. High spatial resolution and high CNR are the main advantages of QISS MRA and these technical features allow reliable detection of small arterial renal vessels. Accordingly, the diameter of the renal artery could be measured accurately with QUISS MRA and, in this analysis, no significant differences were found compared to CTA as reference standard. The diameter of the left and renal artery was comparable to previously published larger patient collectives [25].

Another advantage of sagittally acquired slices is the ability to enlarge the FOV without increasing measurement time. This is of clinical relevance since some aberrant renal arteries can be also located near the aortic bifurcation. Previous studies with axially acquired images had to limit the number of slices to accomplish a reasonable acquisition time and were, consequently, not able to detect all aberrant renal arteries due to the limited FOV [18]. The acquisition time in our study was comparable to other non-enhanced MRA studies. Gietzen et al. reported a measurement time of around 5 min, which is similar compared to this study (left and right kidney) [26]. Due to the older patient cohort in this study, the duration of breath-holding was limited, lengthening acquisition times compared to studies with healthy, young participants [9].

### 4.1 Study limitations

This retrospective study has several limitations. First, the limited number of participants and the elderly patient cohort with corresponding co-morbidities may limit the comparability to studies with healthy participants. Second, assessment of renal artery stenosis was not feasible in this study due to slice orientation of the acquired QISS MRA data. Further studies in a larger patient cohort are warranted to investigative the role of QISS MRA for the detection of renal arterial stenotic disease.

## 5 Conclusions

The results of this retrospective study demonstrated that QISS MRA is suitable and diagnostic for assessing the renal arterial vasculature. QISS MRA offers comparable diagnostic confidence and image quality to CTA. Further, QISS non-contrast MRA demonstrates excellent diagnostic accuracy in detecting anatomical variations of branching patterns of the renal arterial vasculature. In summary, QISS MRA seems to be a robust non-contrast MRA approach for the evaluation of the renal arterial vasculature in daily clinical practice.

## CRediT authorship contribution statement

**Sasan Partovi:** Writing – original draft, Validation, Supervision, Project administration, Methodology, Formal analysis, Data curation, Conceptualization. **Patrick Ghibes:** Writing – original draft, Visualization, Validation, Software, Resources, Project administration, Methodology, Investigation, Formal analysis, Data curation, Conceptualization. **Petros Martirosian:** Writing – review & editing, Supervision, Software, Resources, Methodology, Conceptualization. **Florian Hagen:** Writing – review & editing, Validation, Project administration, Methodology, Data curation, Conceptualization. **Konstantin Nikolaou:** Writing – review & editing, Validation, Supervision, Project administration. **Stephan Ursprung:** Writing – review & editing, Software, Project administration, Formal analysis, Conceptualization. **Abraham Levitin:** Writing – review & editing, Supervision, Project administration, Methodology, Conceptualization. **Daniel Raskin:** Writing – review & editing, Methodology, Conceptualization. **Levester Kirksey:** Writing – review & editing, Supervision, Project administration, Conceptualization.

## Ethical statement

This retrospective HIPAA-compliant study was approved by the local institutional review board (approval number 297/2023BO2). The requirement for informed consent was waived.

## Funding

*This research did not receive any specific grant from funding agencies in the public, commercial, or not-for-profit sectors.*

## Declaration of Competing Interest

The authors declare that they have no known competing financial interests or personal relationships that could have appeared to influence the work reported in this paper.
